# Exploring rare coumarins biosynthesis by *Gloeophyllum trabeum*: an integrated computational genomics, molecular docking and spectroscopic approach

**DOI:** 10.1007/s11274-026-05181-9

**Published:** 2026-08-14

**Authors:** Suhad A. A. Al-Salihi, Trong Tuan Dao, Saipul Maulana, Andy M. Bailey

**Affiliations:** 1 Department of Biotechnology, College of Applied Sciences, University of Technology, Baghdad, Iraq; 2 School of Pharmacy, University of Medicine and Pharmacy, Ho Chi Minh City, Vietnam; 3 Department of Pharmacy, Faculty of Mathematics and Natural Sciences, Universitas Tadulako, Kota Palu, Indonesia; 4 School of Biological Sciences, University of Bristol, Bristol, UK

**Keywords:** Antimicrobials, Brown-rot Basidiomycete, Polyketides, Isocoumarin, Molecular docking, Genomic prospecting

## Abstract

The sustained rise of antibiotics resistance demands the discovery of novel compounds to enhance current treatment efficiency and decrease the spread of resistant pathogens. *Gloeophyllum trabeum* is a common wood-decaying basidiomycete fungus, however its capability for production of specialised metabolites remains poorly understood. We integrated spectroscopic analysis, computational genomic, and molecular docking to explore isocoumarin biosynthesis of this mushroom. Using plate-based overlay bioassays we showed that *G. trabeum* is antagonistic against three microbes: *Escherichia coli*, *Saccharomyces cerevisiae*, and *Bacillus subtilis*, with most effect against *B. subtilis*. The culture filtrates of *G. trabeum* were analysed by LCMS, with four products being further characterised by 2-D NMR, leading to the characterization of four rare coumarin-based polyketides: compound 1(tentatively, hydroxylated naphthoquinone derivative), compound 2 (oospoglycol), compound 3 (oosponol), and compound 4 (Naphthazarin-6,7-dihydrodiol (FD48)). Notably, both oospoglycol and oosponol exhibited antimicrobial activity against *B. subtilis*. Genome mining and annotation of *G. trabeum* predicted several biosynthetic gene clusters (BGCs), including clusters associated with isocoumarin-derived compounds, which likely corresponds to the newly identified metabolites. The docking analysis in its turn identified oosponol as the most promising lead compound, exhibiting exceptional affinity for UspA1 of *Moraxella catarrhalis*, while oospoglycol showed broader antimicrobial potential through interactions with multiple targets, highlighting UspA1 as a key candidate for further future validation. Given that oosponol derivatives are commercially unavailable, due to the high costs associated with their production and purification, therefore characterising these compounds in *G. trabeum* considered an important scientific progress, as *G. trabeum* represents the only species from the Gloeophyllales order with available whole-genome sequences. Characterizing these unique compounds and their putative BGCs in *G. trabeum* boost the potential for high-yield, rapid cost effect production and development of novel antibiotics through synthetic biology and pharmacophore modelling.

**Supplementary Information:**

The online version contains supplementary material available at https://doi.org/10.1007/s11274-026-05181-9.

## Introduction

Natural metabolites play pivotal role in drug discovery, with actinomycetes and ascomycetes being widely investigated for antimicrobials production (Yurchenko et al. 2025). In contrast, basidiomycetes were overlooked, due to their challenging cultivation, despite their remarkable chemo-diversity and longstanding practise in traditional therapies. Progressive advancement in genome mining coupled with synthetic biology, supported deeper exploration of these neglected fungi (Qi et al. 2025). Studies have shown that fungal ecological niche contributes largely to its production of bioactive compounds. Saprophytic mycelia for example, compete with other microbes on nutritional resources, while their fruiting bodies interact with soil invertebrates including nematodes and collembola, influencing their chemo-biological defence (Al-Salihi et al. 2025).

Evolutionary, brown rot fungi evolved a decomposition system capable of accessing both lignin and holocellulose, normally inaccessible to other organisms. *G. trabeum* for instance, a key wood-decaying species in forest, specializes in breaking down cellulose derivatives. Unlike white rot fungi that predominantly target lignin, *G. trabeum* utilizes small, diffusible metabolites that penetrate deep into wood, reaching areas inaccessible to other species, highlighting its enzymatic capabilities (Goodell et al. 2020). This specialized mechanism not only enhances the fungus’s ability to thrive in competitive, nutrient-scarce environments but also demonstrate its potential to produce distinct secondary metabolites (e.g. volatile compounds and enzymes) adapted to these ecological challenges (Rasser et al. 2000). Contrasting numerous medicinal mushrooms, no documentation on the traditional medicinal use of *G. trabeum* could be found in the literature, as it is generally considered inedible mushroom. However, its distinctive ecological role and its accessible genomic data (Floudas et al., 2012), has positioned *G. trabeum* as an invaluable resource for the discovery of novel secondary metabolites with potential therapeutic applications.

Whilst ascomycete genomes commonly have gene clusters for biosynthesis of many non-ribosomal peptides, polyketides and terpenes, in basidiomycetes such gene clusters are dominated by terpene production with polyketide synthesis gene clusters rarely being present (Xie et al. 2024). Coumarins are benzopyrone based compounds belong to lactones family which are made via a polyketide pathway. Their backbone mainly consists of a fused benzene and α-pyrone ring system (Zhu and Jiang 2018). Although coumarins are widely regarded as primarily plant-derived secondary metabolites (Al-Salihi and Ford 2023), a significant fraction of the over 1,300 known coumarins have been discovered in microorganisms, with fungi emerging as the most efficient and previously underappreciated source of pharmaceutically significant coumarin-based compounds. Coumarin compounds possess a unique profile of biological activities, acting as antioxidant, antimicrobial, antiviral, anti-inflammatory and antitumor agents (Tsivileva and Koftin 2023). Several biologically active coumarins have been reported from basidiomycetes (Tsivileva and Koftin 2023). Armillarisin A, for example, sourced from the honey fungus *Armillariella tabescens*, has properties of reducing bile duct inflammation and supporting the regulation of liver function (Wang et al. 2007). The antibacterial properties of fungal coumarins has been assessed in edible mushrooms including *Lentinula edodes*,* Pleurotus ostreatus*, and *Ganoderma lucidum*, revealing the presence of coumarin-based compounds in the extract of the fruiting bodies of *G. lucidum* and demonstrated their activity against *Bacillus cereus* (Islam et al. 2015), whilst (Jose and Radhamany 2013) confirmed the correlation between the antioxidant activity of *Macrolepiota mastoidea* and its coumarin content.

In this study, we characterized four rare polyketides belonging to the isocoumarin class, one of which exhibits unique substitution pattern (one phenolic OH at position 7). Among characterised compounds, oospoglycol and oosponol demonstrated antimicrobial activities. By investigating these bioactive metabolites in a species with publicly available genomic data, we pave the way for leveraging genomic database, spectroscopic analyses, synthetic biology, and molecular dynamic platforms for sustainable utilisation of fungal biodiversity to enhance the yield and efficiency of novel antibiotics.

## Experimental procedures

### Chemicals

All Chemicals and organic solvent used in this study were supplied by Fisher Scientific. The *G. trabeum* strain ATCC11539 was generously provided by Dr. David Hibbett, Clark University (USA), and was maintained on PDYA (24 g/L of Potato dextrose broth, 5 g/L of Yeast extract and 15 g/L of agar) plates throughout this investigation.

### Plate-based bioassays

A 6 mm plug of freshly grown *G. trabeum* agar culture was placed centrally on three replicate plates of each of malt peptone glucose agar (MPGA), yeast extract malt (YMGA), potato dextrose agar (PDA), malt extract agar (MEA), yeast malt dextrose peptone agar (YMATA) and potato dextrose yeast extract agar (PDYA). Plates were then incubated for 48 h to support fungal growth before application of the appropriate overlay. Three model microorganisms were selected (*Bacillus subtilis* ATCC6633 as Gram-positive bacterium, *E. coli* DH5 alpha as Gram-negative bacterium, and *Saccharomyces cerevisiae* Y1000 as a fungal eukaryotic species) to cover a broad and balanced biological selection in terms of cell wall structure and organization. For the overlay plates, stocks of *Bacillus subtilis*, *E. coli*, and *Saccharomyces cerevisiae* were prepared following (Al-Salihi et al. 2021). (refer to supplementary data for cultures conditions).

### Fermentation culture and chemical extraction

A 6 mm agar culture plug of *G. trabeum* was inoculated into a 2 L flask containing 1 L of PDY broth and incubated with shaking at 30 °C for 14 days. To extract the metabolites, 1 L of ethyl acetate was added to the culture, and the mixture was homogenized for 30 min at 25 °C. The homogenate was then filtered, the organic layer was separated and dried using anhydrous MgSO₄ and then dried in a rotary evaporator to yield the crude extract (Al-Salihi et al. 2021).

### Metabolite preparation for HPLC-MS analysis

Having shown that *G. trabeum* produces biologically active compounds on PDYA, a larger scale of culture [2 L] was used to support chemical purification and characterisation. The crude extract was subjected to flash column chromatography using various ratios of methanol and deionized water over silica gel RP-C18 (MeOH/H_2_O) (refer to supplementary data for column conditions). Fractions containing target compounds, were further purified using preparative HPLC with fraction collection guided by LC-MS analysis. Data collected using Waters LCMS system comprising of a Waters 2767 autosampler, Waters 2545 pump system, a Phenomenex LUNA C18 column (2.6 μm, 100 Å, 4.6 × 100 mm) equipped with a Phenomenex Security Guard precolumn (Luna C5 300 Å) eluted at 1 mL/min. Detection was by Waters 2424 ELSD and Waters SQD-2 mass detector operating simultaneously in ES + and ES- modes (Kramer and Deming 2010).

### NMR characterization of target compounds

Purified compounds were dried and dissolved in CDCl_3_ for the subsequent NMR analysis using an Agilent VNMRS500 spectrometer, measuring ¹H-NMR, ¹³C-NMR, and coupling constants (J) expressed in hertz (Hz). Two-dimensional NMR techniques (COSY, HSQC, HMBC) were used routinely for the assignment of structures (Sauri, 2026).

### Antimicrobial bioassay of purified compounds

Following compound purification, bioassays were conducted to assess the antimicrobial activity of (compounds 1, oospoglycol, oosponol, and FD48). Each compound was dissolved in methanol to a concentration of 1 mg/mL. The protocol described in the supplementary data, was then employed to prepare three replicate overlay plates of *B. subtilis*, a 5 mm central well was created in each plate, and a 100µL of an appropriate dilution of the purified compound was added to the well. Plates were then incubated for 24 h at 28 °C before measuring the resulting zone of clearance.

### Molecular interaction modelling

#### Molecular docking

To address the limited number of technical and biological replicates during the antimicrobial bioassay, and to investigate the relationship between the biological activity and protein binding, compounds representing inactive (compound 1), semi-active (oospoglycol), and active (oosponol) were further evaluated against a broader range of microbial species (including *Burkholderia pseudomallei*,* Escherichia coli*,* Moraxella catarrhalis*,* Neisseria meningitidis*,* Staphylococcus aureus*,* and Bacillus subtilis*) using molecular docking analysis. Given the reported inhibition activity of isocoumarin against enzymes involved in DNA and membrane synthesis (Ashraf et al. 2014), highly selective antimicrobial targets for drug characterisation and development were selected for our docking analysis, considering their known role in bacterial survival, and the absence of such pathways in human. Native inhibitors including triclosan, ceftobiprole, berberine and lumacaftor, provided validated benchmarks for evaluating our tested isocoumarin compounds (Sivaraman et al. 2004).

The comparative analysis included several enzymes relevant to the microbial survival and drug resistance (e.g. Enoyl-Acyl Carrier Protein Reductase (MurG), D-sedoheptulose 7-phosphate isomerase (GmhA), Filamenting Temperature-Sensitive Mutant Z (FtsZ), Enoyl-acyl carrier protein reductase I (FabI), Ubiquitous Surface Protein A1 (UspA1), Factor Essential for Methicillin Resistance (FemX), sulfur mobilization protein (SufS), from both Gram-positive and Gram-negative bacteria (refer to Table S1 for further details on the target proteins). The three-dimensional (3D) crystal structures of the bacterial protein targets were retrieved from the Research Collaboratory for Structural Bioinformatics (RCSB) (accessible via: https://www.rcsb.org/ ). Subsequently, each receptor structure was prepared using the Molecular Operating Environment (MOE) 2020 software suite (ULC 2020**)**. The initial preparation involved a refinement process in which hydrogen atoms were added and the protonation states of residues were optimized. Additionally, energy minimization of each macromolecule was performed using the AMBER99 force field until a stable conformation was achieved. The binding pocket for each specific receptor was defined by the coordinates of its co-crystallized native ligand, or reported in previously published literature (Uddin et al. 2025**)**. In cases where no co-crystallized ligand was present, specifically for *E. coli* FtsZ and *S. aureus* FemX, a computational prediction of the binding pocket was necessary using the DogSiteScorer (accessible via the Proteinsplus platform: https://proteins.plus/help/poseview) which identifies potential cavities on the protein surface and evaluates their suitability for ligand binding. The selection of the most probable active site based on the DrugScore, which evaluates properties such as pocket size, hydrophobicity, and potential for interaction. Consequently, the pocket exhibiting the highest DrugScore for each of these two receptors was selected as the target for docking simulations, as this value indicates a high probability of being a druggable site. The selected compounds (compound 1, oospoglycol and oosponol) were initially constructed as 2D structures using ChemDraw 19.1 software and subsequently imported and optimized into 3D into the MOE database. This process involved the addition of both polar and nonpolar hydrogen atoms, followed by a conformational search and energy minimization using the MMFF94x force field to obtain the most stable, low-energy conformer for each ligand. A set of known inhibitors or relevant compounds was included as positive controls for each target. These control ligands {5-(4-amino-2-methylphenoxy)-2-hexyl-4-hydroxy-1-methylpyridinium (ID = 73437603), 1-hydroxy-2,3,1-benzodiazaborinine-2(1 H)-carbothioamide, Triclosan, Lumacaftor, (2~{R})-3-methyl-2-[(~{E})-[2-methyl-3-oxidanyl-5-(phosphonooxymethyl)pyridin-4-yl]methylideneamino]-3-sulfanyl-butanoic acid)} underwent the same preparation protocol as the three selected metabolites.

#### Molecular dynamics

To assess the ligand-receptor binding stability for the selected protein targets with our compounds, Molecular dynamic simulation (MD) was used. The analysis carried out for 100 ns via the AMBER 2024 simulation package under the following conditions: time step of 2 fs and periodic boundary were applied. For the long-range electrostatic interactions, the Particle Mesh Ewald (PME) was used, while the SHAKE algorithm was used to constrain the covalent bonds (hydrogen atoms). The Root Mean Squar deviation (RMSD) of the ligand was subsequently calculated throughout the simulations run time using the CPPTRAJ module (Roe and Cheatham 2013).

#### Bioinformatic analysis of secondary metabolite gene clusters

The whole genome of *G. trabeum* was first retrieved from the JGI and NCBI websites (accession no = AFVP00000000.1 and taxonomy ID = 670483) followed by an initial analysis through the antiSMASH platform (Blin et al. 2023) for prediction of the types of BGCs. This analysis was further refined by inclusive manual annotation using Softberry, Artemis and Local BLAST search (Softberry Inc., 2026; Carver et al. 2012; Camacho et al. 2009). To further support the gene cluster predictions and explore any potential function/sequence homology with experimentally characterized bioactive PKS, a search of MiBig (Zdouc et al. 2025) was performed. Additionally, a phylogenetic analysis was performed using the Mega11 software (Tamura et al. 2021), to assess the evolutionary relationship of the *G. trabeum* candidate PKSs with different characterized PKSs known to participate in the biosynthesis of biologically active polyketide metabolites, providing additional evidence for the identification and functional assignment of the biosynthetic gene(s) potentially responsible for oosponol production in *G. trabeum*. Method and model description are included in Table 1.

**Table 1:** The parameters used for constructing the maximum likelihood phylogenetic tree

Option	Statistical method	Test of phylogeny	No. of bootstrap replications	Substitutions type	Model	Rates among sites	No. of discrete Gamma categories	Gaps missing data treatment	Cite coverage cutoff%	ML heuristic method	Initial tree for ML	Branch swap filter	No. Of thread
**Setting**	Maximum likelihood	Bootstrap	100	Amino acid	WAG model	Gamma distributed	5	Partial deletion	95	Nearest Neighbour Interchange (NNI)	Neighbour Joining	Strong	5

## Results

### Assessment of *G. trabeum* antagonistic activity

**Fig. 1:**
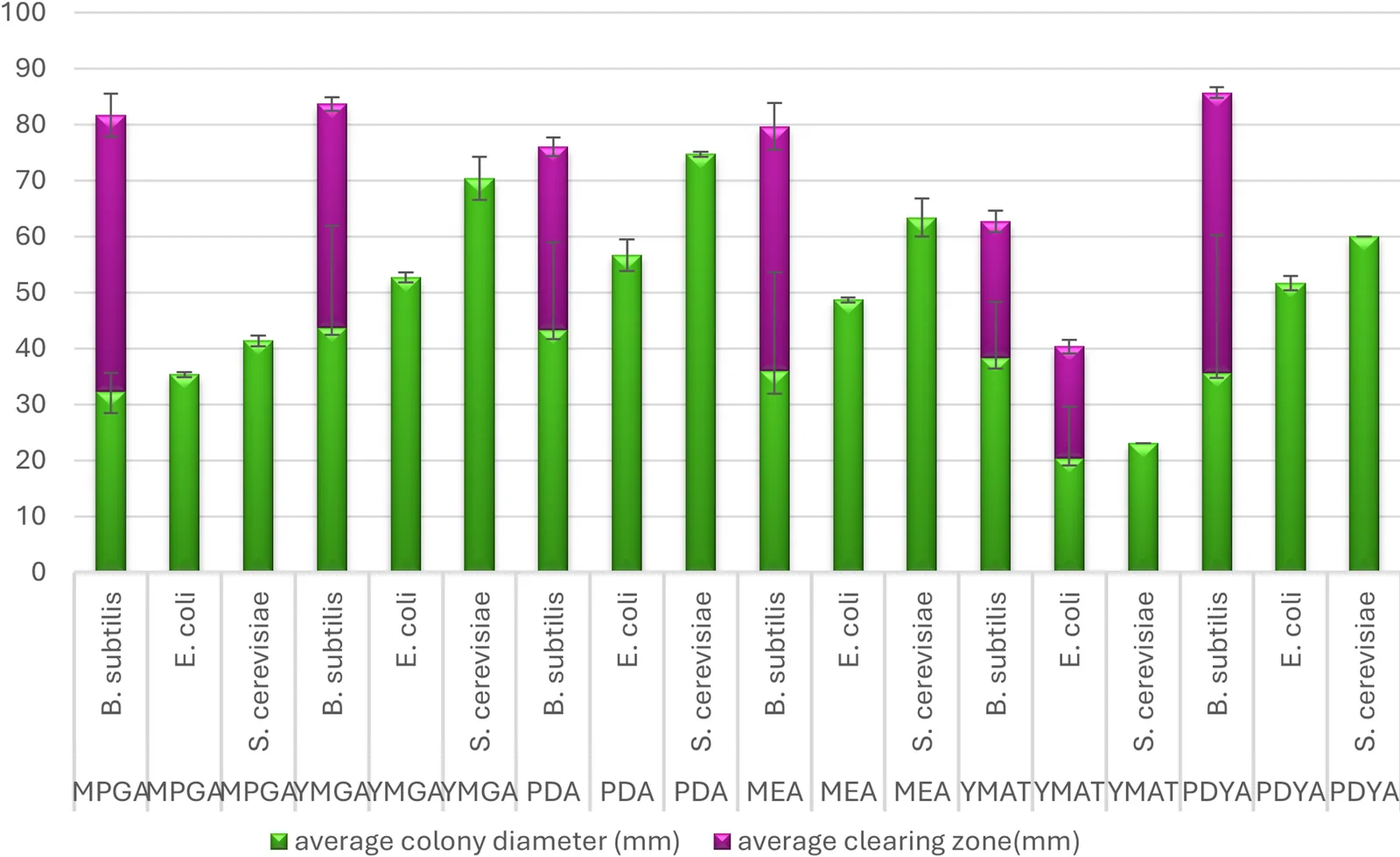
Bioassay test to evaluate the antimicrobial activity of *G. trabeum* growing on different media against *B. subtilis*, *E. coli* and *S. cerevisiae*. MPGA = malt peptone glucose agar, YMGA = yeast extract malt agar, PDA = potato dextrose agar, MEA = malt extract agar, YMAT = yeast malt dextrose peptone agar, PDYA = potato dextrose yeast extract Agar. Error bars indicate the standard deviation of three biological replicates for both fungal colony diameter and inhibition zone diameter

The preliminary investigation of *G. trabeum* antimicrobial activity against panel of pathogenic microbes confirmed its ability to produce biologically active substances. Microbial susceptibility differed between species, and between different fungal growth media. Generally, *B. subtilis* was the most susceptible microbe towards *G. trabeum* mycelial growth, whereas *S. cerevisiae* and *E. coli* showed less susceptibility. The highest zone of inhibition of *B. subtilis* was achieved when *G. trabeum* was grown on PDYA plates (Fig. 1), hence this medium being used for preparative-scale culture. The only medium where there was detectable inhibition of *E. coli* was YMAT medium.

### Compounds purification, characterisation and preliminary bioactivity test

**Fig. 2:**
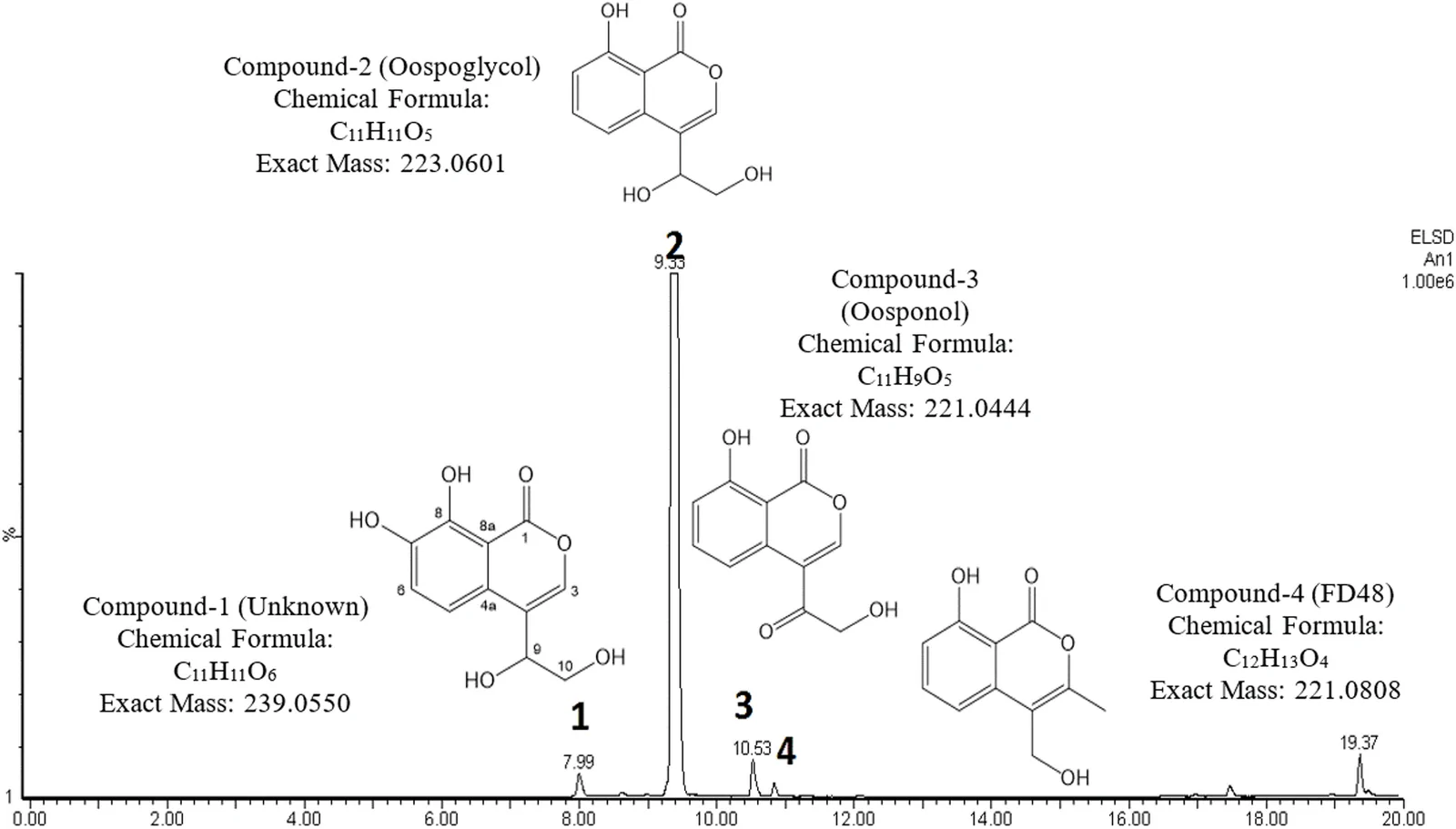
HPLC profile and chemical structure of four characterised compounds from *G. trabeum* culture

Flash chromatography was undertaken using various ratios of methanol and deionized water to purify active metabolites from the crude ethyl acetate extracts of *G. trabeum.* Further HPLC and NMR analysis, led to the identification of four coumarin-like compounds: compound 1(tentatively, hydroxylated naphthoquinone derivative), compound 2 (oospoglycol) (Sonnenbichler et al., 1997), compound 3 (oosponol) (Kovacs et al., 1997), and compound 4 (Naphthazarin-6,7-dihydrodiol (FD48)) (Sonnenbichler et al., 1989), with compound **1** displaying a novel chemical structure (Fig. 2). Refer to table S2 and Figs. S1–S22 in the supplementary data for further details on UV/vis [DAD], [±] ESI-MS, IR and NMR spectra for the isolated compounds.

The overlay bioassay of these compounds, at a concentration of 1 mg/mL, revealed that oospoglycol and oosponol showed activity against *B. subtilis* (Table 2).

**Table 2:** Overlay bioassay test for the four purified compounds from G. trabeum*

Microorganism: *Bacillus subtilis*	
Compound (1 mg⁄ml)	Clearing zone (mm)
New compound (compound 1)	No clearing zone
Oospoglycol (compound 2)	12 (faint clearing zone)
Oosponol (compound 3)	16
FD-48 (compound 4)	No clearing zone

* These results represent one replicate due to compounds limitation

### Molecular interaction modelling

**Fig. 3:**
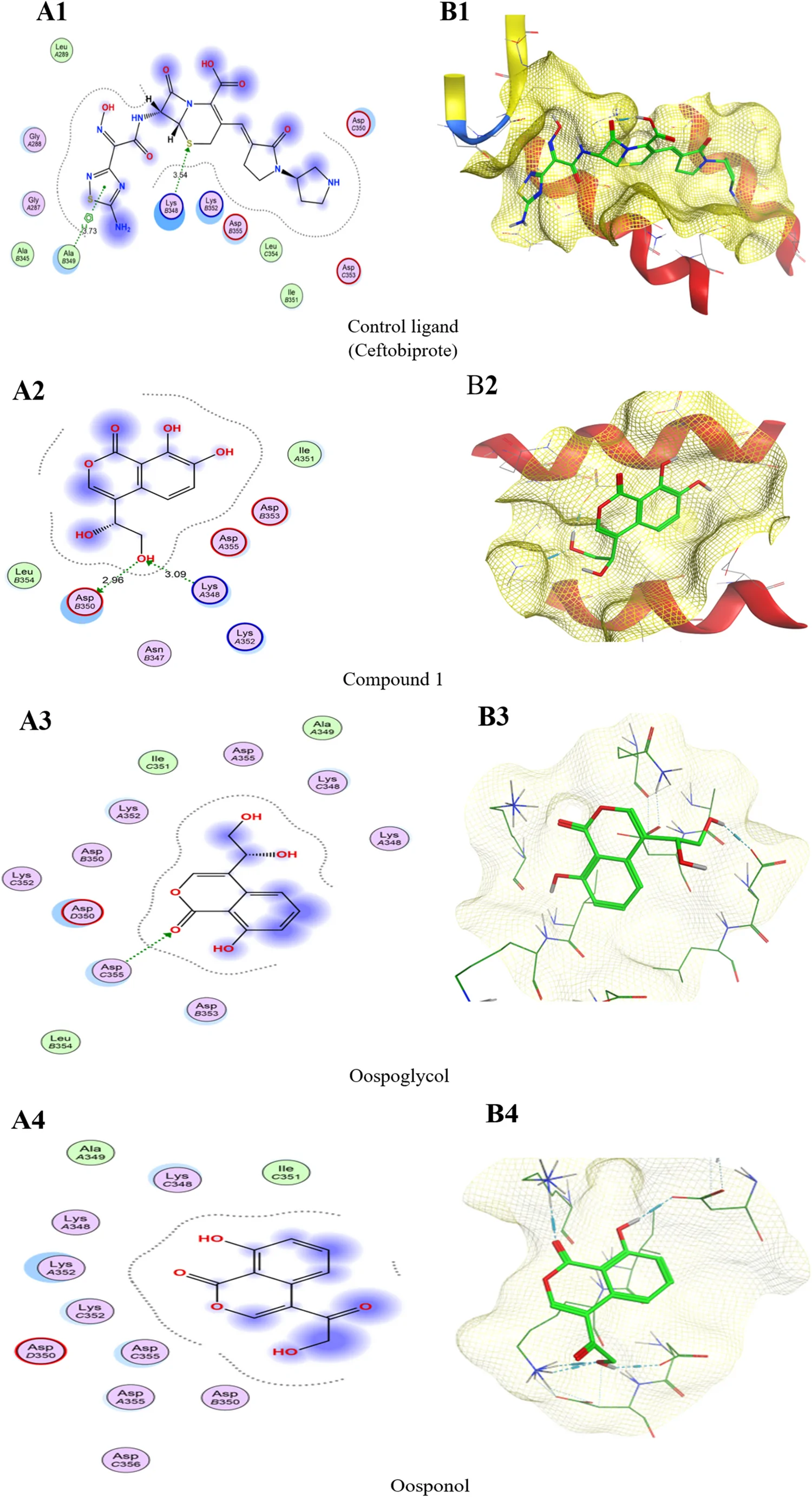
Illustrates the binding orientations and key molecular interactions between native ligand (Ceftobiprole) and candidate compounds with the target protein (UspA1) binding pocket. The left panel (A) shows the two-dimensional (2D) interaction maps, while the right panel (B) presents the corresponding three-dimensional (3D) binding poses within the protein active site. A1–B1 represent the control ligand; A2–B2 represent Compound 1; A3–B3 represent oospoglycol; and A4–B4 represent oosponol

**Fig. 4:**
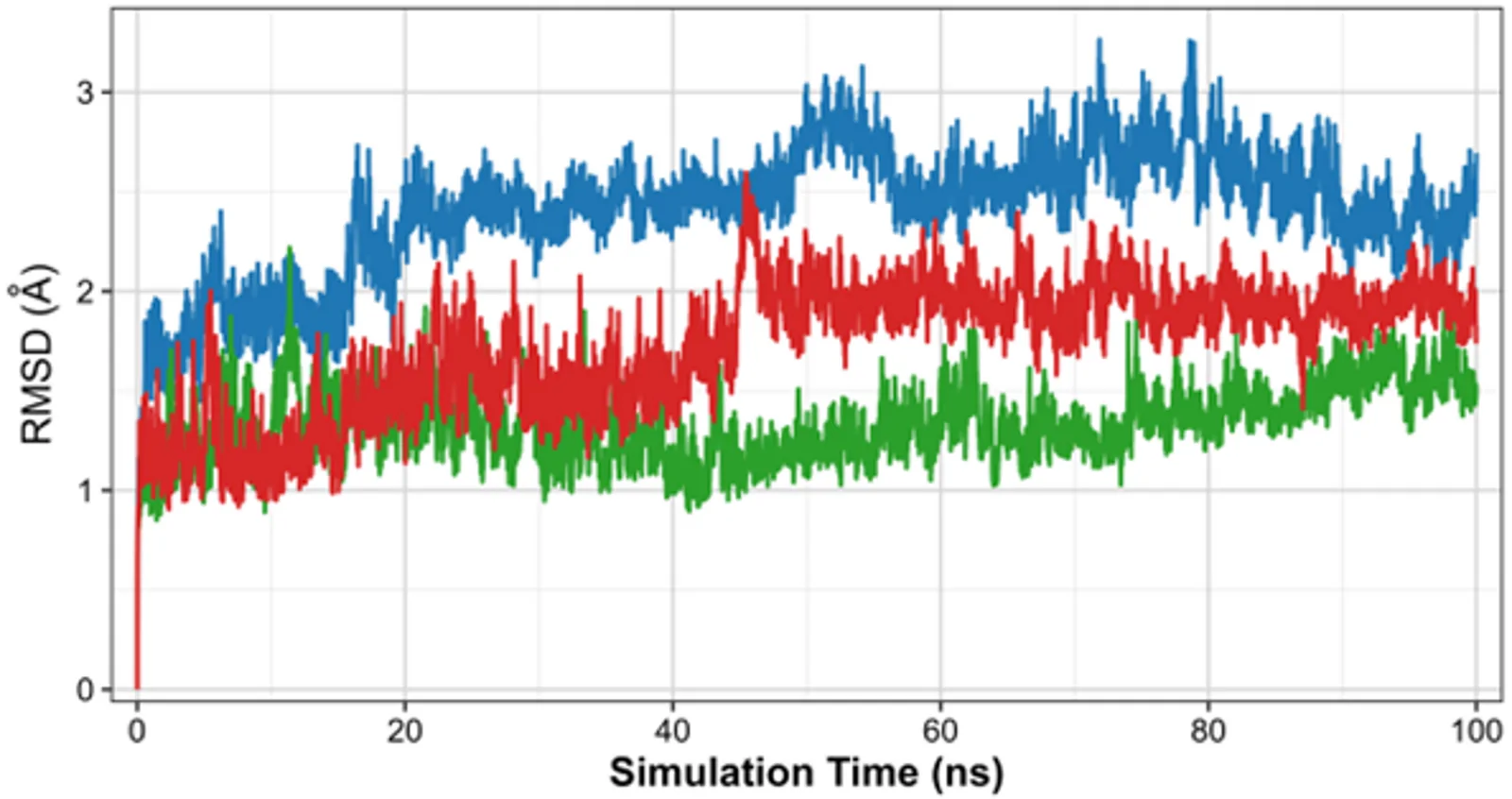
RMSD profiles of three selected receptor–ligand complexes during a 100 ns molecular dynamics simulation, demonstrating the structural stability and the conformational fluctuations of each complex over time. The RMSD values (Å) were calculated relative to the initial structure. The colored traces represent the following complexes: blue; *M. catarrhalis* FabI (PDB ID: 7CMI)-ligand compound 1, green; *M*. *catarrhalis* UspA1 (PDB ID:3NTN)-ligand oospoglycol, red; *M. catarrhalis* UspA1 (PDB ID:3NTN)-ligand oosponol

The three selected compounds were tested against *s*ix pathogenic microorganisms, with two target proteins for each bacterium, except for *N. meningitidis* and *B. subtilis* for which only one target protein could be optimised for docking investigation (due to the large size of protein and lack of active sites). Generally, the docking scores indicated that the three compounds exhibited selective target preferences, with oospoglycol and oosponol demonstrating particularly strong affinity toward one targeted protein (UspA1) from *M. catarrhalis* (Fig. 3). Starting with *B. pseudomallei*, where the reference ligands exhibited strong binding affinities to both receptors MurG (-7.0915 kcal/mol) and GmhA (-6.3196 kcal/mol) outperforming all test compounds. Whereas for the second examined bacterium (*E. coli*), the scenario was slightly different, as the reference ligand showed competitive interaction with FabI receptor compared to the three test compounds, however for the second target protein (FtsZ) oospoglycol exhibited the highest score (-5.7261 kcal/mol) followed by oosponol (-5.5290 kcal/mol). For the third bacterium (*M. catarrhalis*) protein targets (FabI and UspA1); the binding affinities were relatively different, as both displayed moderate bindings (-5.4 to -5.6 kcal/mol) in terms of FabI receptor, and outstanding interactions (-13.552 and − 11.713 kcal/mol) in terms of UspA1 receptor, could be shown by oosponol and oospoglycol. The fourth tested bacterium was *N. meningitidis*, for which, all tested compounds showed weaker affinities (-5.3181 to -5.5706 kcal/mol) compared to their native ligand (-5.9342 kcal/mol). In the fifth bacterium (*S. aureus*), our compounds showed variable affinities with no competitive inhibition potential towards both selected target proteins (FabI and FemX) as for FabI oospoglycol (-5.4800 kcal/mol) and compound 1(-5.6214 kcal/mol) showed slightly better affinities than oosponol (-5.3962 kcal/mol), however still below the native ligand’s score (-5.8284 kcal/mol), while for FemX, the rank was oosponol (-5.7775 kcal/mol), followed by oospoglycol (-5.7059 kcal/mol) and Compound 1 (-5.6206 kcal/mol), and again lower than their reference ligand (-7.0188 kcal/mol). The final selected bacterium was *B. subtilis*, for which all three test compounds showed weaker interactions with scores ranging from − 5.4501 to -5.6409 kcal/mol in comparison to the reference ligand’s binding affinity (-8.5319 kcal/mol) (refer to supplementary data Figs. S23–S32 for ligands and protein receptors interactions). These results were further validated by molecular dynamics (MD) analyses, which showed that the majority of protein-ligand complexes maintained RMSD values of approximately 1.5-3.0 Å throughout the simulation, indicating stable binding and overall conformational stability (refer to Fig. S33 in the supplementary file for detailed description). As presented in Figure 4, compound 1-FabI complex, exhibited an initial increase in its RMSD during the first 15-20 ns, before its final stabilisation between 2.3 and 2.8 Å, whereas the equilibration phase of the oospoglycol–UspA1 complex was reached earlier with a maintained RMSD profile ranging from 1.0 to 1.6 Å. Similarly, the oosponol–UspA1 complex displayed an initial increase followed by moderate structural fluctuation around 1.8–2.1 Å.

### Phylogenetic analysis of the predicted PKS enzymes

A phylogenetic tree was constructed based on the eight putative PKS genes predicted in *G. trabeum*, and a subset of functionally characterised polyketide synthases (PKSs) involved in the biosynthesis of diverse polyketide like antibiotics from different backbones (e.g. non-reducing polyketide synthase (NR-PKS), highly reducing polyketide synthase (HR-PKS) and hybrid polyketide synthase-non ribosomal (HPKS-NRPS) sourced from the MIBiG database. The generated Maximum Likelihood tree organised the enzymes into five main clades, each involving 2–5 subclades, reflecting the evolutionary relationship and homologies between the predicted *G. trabeum* PKSs and experimentally characterised PKS with distinct domain architecture and biosynthetic function. Our predicted *G. trabeum* PKSs (denoted with red stars), were distributed across four of the five generated clades, indicating their functional diversity. The first clade (clade 1), grouped enzymes of hybrid PKS-NRPS backbone, associated with the biosynthesis of biological active compounds, including, equisten, fusarins, lovastatin and related metabolites. The second clade (clade 2), comprised predominantly HR-PKS, where several enzymes from the predicted *G. trabeum* PKSs clustered closely with two biosynthetic genes involved in strobilurin and polyene biosynthesis, suggesting their potential role in the production of structurally related compounds. In contrast, clade 3, contained only one predicted *G. trabeum* PKS (PKS 8 − 1), which grouped with NR-PKSs that are responsible for the biosynthesis of alternariol and citrinin biosynthesis. In the fourth clade (clade 4), more heterogenous collection of PKSs correlated to the biosynthesis of structurally diverse compounds, including necroxime, lucensomycin, spicicostatin and erythronolide were grouped with two predicted of *G. trabeum* PKSs. As for the fifth clade (clade 5), it consisted mainly of bacterial based PKSs associated with the biosynthesis of antimicrobial compounds such as streptoketide, pateamine and sorangicin, therefore no predicted *G. trabeum* PKS located in this clade (Fig. 4). Further comparative domains analysis of PKS8-1, revealed its high conservation and syntenic with alternariol PKS which involved the following domains SAT, KS, MAT, PT, and ACP (Fig. S34) 

### Genome mining and BGCs characterization

Previous work by (Kovács, and Sonnenbichler, 1997) in another related species (*G. sepiarium*), proposed that oosponol and oospoglycol were PKS-derived compounds. Utilising the 13-C labelled acetate, they further revealed that the extra carbon [methylene carbon [–CH₂–]] most likely comes from the S- adenosyl methionine, (post-PKS methylation). To confirm this genetically, we used the publicly available genome sequences of *G. trabeum*. The initial search of *G. trabeum* SM core enzymes on JGI platform predicted the following core enzymes: eleven NRPS-Like, thirteen PKS-like (of which 8 were within BGC), and seven terpene cyclase. On the other hand, FungiSmash predicted the following BGCs: nine NRPS, six T1PKS, three hybrid T1PKS-NRPS, twenty-two terpene like BGCs (refer to table S3 supplementary data).

**Fig. 5:**
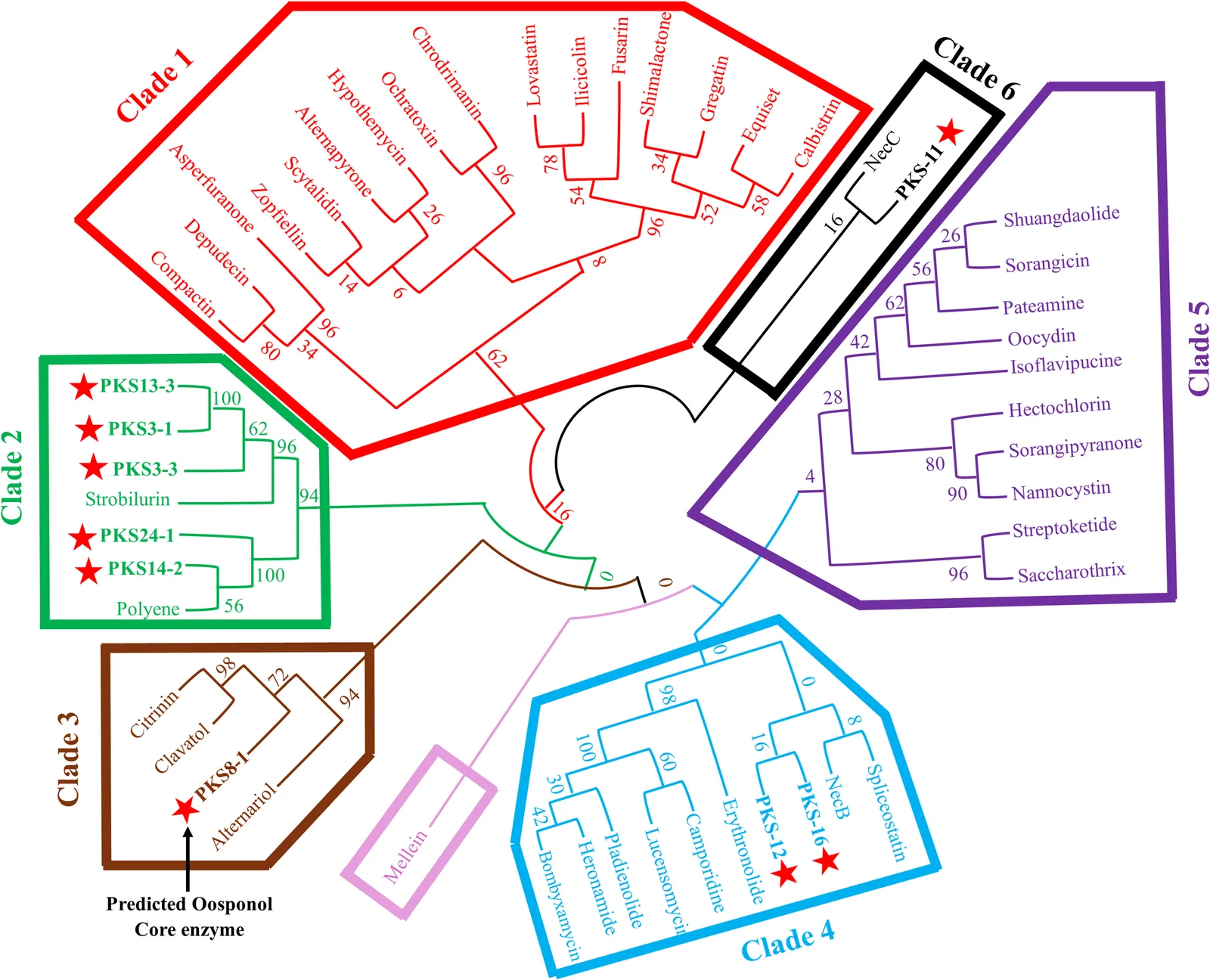
Maximum-likelihood phylogenetic tree of predicted PKS core enzymes from *G. trabeum* and previously characterised fungal PKSs. Six major clades are highlighted using coloured geometric outlines (red, green, brown, light blue, purple, and light pink). Red asterisks indicate PKS predicted from *G. trabeum*, whereas other labels represent experimentally characterised enzymes annotated with either their associated polyketide products or biosynthetic functions. Numbers at internal nodes indicate bootstrap support values (%)

The antiSMASH analysis indicated that none of the predicted T1PKS-like biosynthetic gene clusters harbour methyltransferase-encoding genes within their predicted boundaries (unpublished data). Consequently, a comprehensive functional analysis of the eight predicted PKS clusters (a combination of PKS genes predicted by JGI and antiSMASH) distributed across scaffolds 3, 8, 11, 12, 13, 14, 16, and 24 was carried out using Softberry, Artemis and local blast search to uncover any previously overlocked functional genes. Each annotated BGC encoded at least one PKS or a derived key enzyme within its boundaries. Remarkably, some scaffolds (3, 13 and 16) contained two PKS like enzymes. Interestingly, our analysis revealed that among the eight annotated PKS-like BGCs (refer to fig. S35 supplementary data for clusters details), a potential BGC on Scaffold-8 (spanning the region 1–69,237) is likely responsible to produce coumarin-derived compounds Fig. 5.

## Discussion

The ability of *G. trabeum* was confirmed during the preliminary assessment of its mycelial growth on different media. The susceptibility of tested microorganisms varied not only among species, but also across the used media. The observed limited inhibition of *S. cerevisiae* is probably because it would be less likely to have a metabolite that showed differential activity between fungi (Melvydas et al. 2020). The different media used here would be expected to deliver different compounds, indeed this is in part with the basis of the OSMAC approach to best demonstrate the many different compounds one strain might be able to produce (Gattinger et al. 2023). Moreover, the observed largest clearing zone of the mycelial growth (50 mm) in comparison to oospoglycol (12 mm) and oosponol (16 mm), indicates the presence of additional minor derivatives and/or synergistic effects among metabolites that were not fully characterised during chromatographic purification due to their low abundance and poor separation.

Although oospoglycol and oosponol, were previously characterised from *Gloeophyllum abietinum* by Kovács, and Sonnenbichler in 1997, our 2-dimensional NMR spectroscopic analysis, enabled solid structural characterization by confirming the presence of additional substituent groups (–C(= O)–O–), providing a comprehensive background for four rare coumarins, with one of them (compound 1) being the first reported occurrence of this molecule in *G. trabeum*. Furthermore, Kovács, and Sonnenbichler (1997), also reported the antimicrobial activity of oosponol against several pathogenic microbes including *E. coli*, *Pseudomonas aeruginosa*, *S. aureus*, as well as the fungal pathogen *Heterobasidion annosum*. They further demonstrated that the antibiotic activity of this compound was primarily attributed to its potent inhibition of RNA and protein synthesis in target cells (Sonnenbichler and Kovács 1997). They concluded that the toxicity of these compounds linked to the presence of an acetyl group at position 4 of the isocoumarin ring, and that the phenolic (C8) and aliphatic (C10) hydroxy groups appear to have minimal influence on the antibiotic effects, although they do enhance biological activity to some degree (Sonnenbichler and Kovács 1997). Another group research (Dietrich et al. 1994), highlighted the high chemical reactivity of oosponol, supporting the idea that the vinyl acid anhydride group, with its strong nucleophilic reactivity, is key to the compound’s biological activity. Using technique like H-1 and C-13 NMR spectroscopy, they suggested that those compounds bind non-specifically to the nucleophilic agents in the cell, including -SH or -NH groups in the amino acid side chains of numerous enzymes, thereby disrupting essential cellular pathways (Dietrich et al. 1994). To complement these findings, we performed molecular docking analysis for the three selected compounds including oospoglycol and oosponol. The outcome of this analysis demonstrated that the biological activities of the three investigated compounds (including oosponol) depend not only on their binding affinities but also on the nature of their molecular interactions established within the active sites of the selected target proteins. Strong hydrogen-bonding networks for example, are known to contribute to ligands stability and binding persistence, particularly those involving conserved backbone (Matta et al. 2006). This was exemplified in the strong interaction of the reference ligands against MurG and GmhA from *B. pseudomallei*, whereas weaker affinities displayed by the three compounds (oospoglycol, oosponol, and compound 1), indicating lower efficiency in disrupting cell wall and lipopolysaccharide biosynthesis pathways in this bacterium. Side chain mediated interactions can also improve ligands specify and molecular recognition irregularly (Liu et al. 2018). This behaviour was observed with the FabI receptors from *N. meningitidis*,* M. catarrhalis*,* S. aureus* and *E. coli*, where the tested compounds demonstrated comparable moderate affinities without exceeding the reference inhibitors scores. FabI represents an essential protein for the bacterial fatty acid synthesis, and its inhibition requires an interaction between the catalytic residues and the NAD+ binding region (Heath et al., 2004), therefore, the inability of some of the tested compounds (except oospoglycol) to surpass the reference ligand affinities, suggest their limited efficacy in binding to the catalytic residues of FabI. Additionally, targeted interaction with the functional amino acids can efficiently block enzymatic response. For example, interactions involved with the conserved residues (e.g. Lys 164) in FabI, are generally responsible for the inhibitor potency of the binding ligand (Ribeiro et al. 2020). Similar principle can be applied for the notable binding affinity of oospoglycol against FtsZ receptor in *E. coli*, which exceeded the native ligand. This favourable performance of oospoglycol suggests its selectivity towards inhibition of cell division, and an attractive antibacterial agent, as FtsZ plays an important role in Z-ring formation and bacterial cytokinesis (Ebrahimi and Samanta 2023; Hurley et al. 2016). Hydrophobic interactions in their turn can significantly strength ligands binding through van der Waals contacts, however, unbalanced hydrophobicity can compromise both target selectivity and aqueous solubility (De Simone et al. 2013). This phenomenon was evident in the interactions of the control ligands (e.g. berberine and lumacaftor), as part of their affinity was originated from extensive hydrophobic contacts with their respective binding pockets. However, a combination of hydrophobic, electrostatic and polar interactions is a prerequisite for optimal inhibitors. Notably, oosponol and oospoglycol demonstrated the most robust interaction with the adhesion-associated receptor UspA1 of the *M. catarrhalis* among all docked complexes, indicating their exceptional binding abilities, and their potential antibacterial mechanism which is mediated through UspA1 inhibition. Given that, UspA1, is a principal virulence factor for the host cell attachment and bacterial colonization, its alteration would inhibit the survival of *M. catarrhalis* (Tan et al. 2005). On the other hand, the three compounds displayed moderate affinities against FemX of *S. aureus* and SufS of *B. subtilis*. FemX is essential protein for cell wall assembly (Benson et al. 2002), while SufS plays a key role in sulfur and iron-sulfur biogenesis (Outten et al., 2015). This relatively weak interaction observed for the three compounds, indicates that the inhibition mechanism of these compounds is different. Furthermore, while oosponol exhibited the largest clearing zone in the in vitro antibacterial assay, its docking scores were slightly weaker than oospoglycol. This discrepancy between the in vitro antibacterial assay and the docking results, indicates that these compounds may involve distinct inhibition pathways other than those predicted within our molecular docking analyses.

**Fig. 6:**
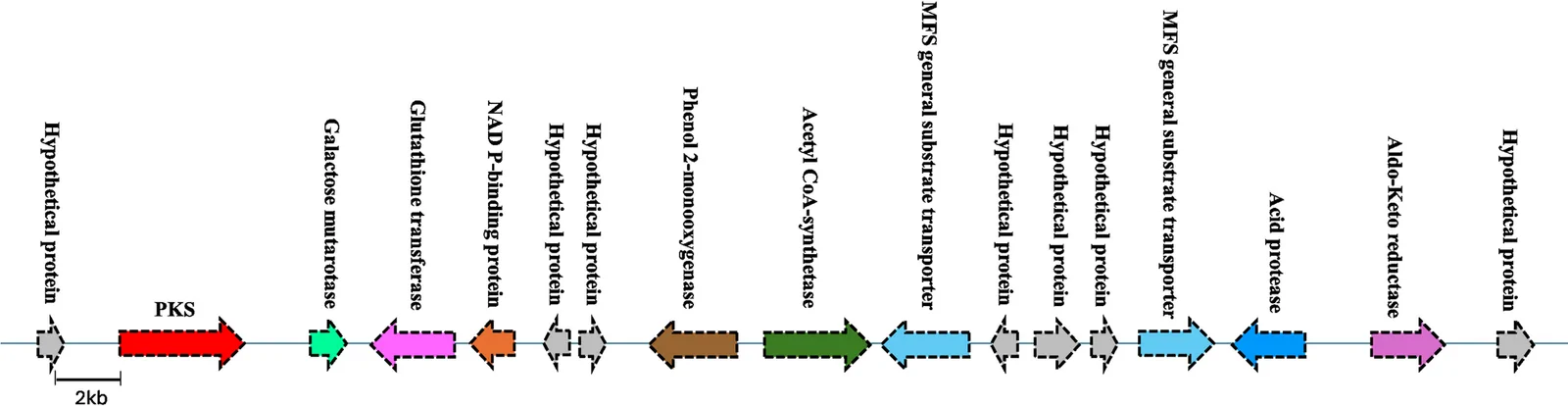
Predicted BGC for the biosynthesis of oosponol and its derivatives

Moreover, a range of RMSD values (~ 1.5–3.0 Å), were maintained for most complexes, suggesting relatively stable binding and conformational rigidity over time (Fig. 6). The *M. catarrhalis* – UspA1 complex for example, exhibited the highest binding stability, with consistent low RMSD and minimal fluctuation (~ 1.0–2.2 Å), implying strong stabilised mode of binding. Similarly, relative stable RMSDs were observed in the *E. coli* – FabI and *S. aureus* – FabI systems with only minor fluctuations. In contrast, the *B. pseudomallei–MurG* and *S. aureus–FemX* complexes, demonstrated lower stability, as their RMSD values stabilised around ~ 3.5–4.5 Å, suggesting tolerable ligand adjustment or greater flexibility. The remaining receptor complexes, displayed notable higher fluctuations, ranged from ~ 2.0 to 5.0 Å, reflecting an increased ligand mobility, weaker interaction and possible multiple binding conformations. This observed behaviour aligns well with previous molecular dynamic reports, especially for the FtsZ target, where the RMSD values exceed 3.5 Å, due to its intrinsic flexibility and non-ideal binding interactions. Overall, the binding stability ranking (UspA1 > *MurG* and *FemX* > FtsZ), is in consistent with the reported literature benchmarks ~ 2.5 to 5.0 Å, where stable, moderate and instability or conformational heterogeneity is reflected (Sasidharan et al. 2023**)**. From drug optimization perspective, the UspA1 protein represents the most promising target for further drug development, followed by *MurG* and *FemX*, which may require minor structural modifications to enhance its binding rigidity. On the other hand, the remaining proteins are likely requiring major or extensive ligand modification to improve their stability and interaction through the introduction of hydrogen bonding or electrostatic contacts.

We further supported these structural insight via our phylogenetic analysis, where the predicted *G. trabeum* PKS enzymes were distributed across four different clades. This distribution highlights the functional diversity of its polyketide biosynthetic machinery. Phylogenetic analysis represents a robust technique to predict the function of previously uncharacterised genes because enzymes produce structurally related compounds share conserved domains and evolutionary relationship (Wang et al. 2023; Cox 2023). However, phylogenetic proximities should be interpreted cautiously, as they may only reflect the backbone of the metabolites, whereas the chemical structure of the end product is largely modified by the associated tailoring biosynthetic genes and the overall BGC organisation (Wang et al. 2023). In this context, the localisation of several *G. trabeum* predicted PKSs within clade 2, a clade dominated by HR-PKS including fungal PKS enzymes knowing for their bioactivity (e.g. the antifungal strobilurin and the macrolactone-based molecule, Polyene (Nofiani et al. 2018; Lee et al. 2012), and their relatively high sequence homology [≥ 70%] indicates that these enzymes are closely related to the *G. trabeum* enzymes (e.g. PKS-14-2 and 13 − 3) (Evidente 2024). Likewise, the close clustering of PKS8-1 in clade 3 with functionally characterised NR-PKSs that are responsible for the biosynthesis of aromatic or isocoumarin-based compounds (e.g. such as alternariol, citrinin and clavatol) (Sun et al. 2024; Sasseville et al. 2024; Kamle et al. 2022; Chooi et al. 2015), implies its potential role in the production of aromatic compounds. These predictions consistent with recent studies reporting the evolvement of fungal iterative PKSs into distinct functional groups that correlate with their catalytic pattern, reduction patterns and product scaffold (Cox 2023; Löhr et al. 2023). In contrast, the placement of PKS12 and PKS16 in clade 4 (heterogeneous clade), indicates a potential evolution in these enzymes enabling them to synthesize diverse polyketide like compounds. This functional diversification is reciprocated in fungal PKS development, and is triggered by domain evolution, gene duplication and biosynthesis pathway specialisation (Wang et al. 2023; Cox 2023). Taken together, the absence of *G. trabeum* PKSs from clade 5, a clade domminated by bacterial PKSs, further supports the evolutionary divergence between fungal and bacterial PKSs. Despite that these enzymes originated from a common ancestral PKS, their families have diverged in domain architecture, catalytic characteristics, and biosynthetic mechanisms throughout their evolution (Grininger 2023). Moreover, one of the predicted core enzymes of PKS-based BGCs were placed in separate and more distantly related branches of the tree (e.g. PKS-11 in clade 6). This observed differences in the end products of the clustered enzymes explains the distinct role that these enzymes play in SMs biosynthesis and their natural implications (Elhamouly et al. 2022). Overall, these findings suggest that *G. trabeum* encode structurally diverse PKS repertoires, reflecting its biosynthetic potential for the production of multiple classes of bioactive polyketides, however, complementary investigations including transcriptomics and heterologous expression are required to validate the metabolites biosynthesized by these predicted PKSs (Cox 2023).

Among these compounds, oosponol and oospoglycol are isocoumarin derivatives, originated from the fungus *Gloeophyllum abietinum.* Notably, microbial dehydrogenation of the biologically inactive oospoglycol yields the active oosponol. Consistent with this performance, the chemical synthesis of oosponol starts with 8-hydroxy-isocoumarin-4-carboxylic acid backbone, followed by the addition of an acetyl group (acetylation) to protect the hydroxyl group at C-8 position. This intermediate is subsequently modified through sequential modifications including, condensation with diethyl ethoxymagnesiummalonate, acid hydrolysis and decarboxylation to generate the 4-acetyl-8-acetoxy-isocoumarin. Thereafter, the introduction of acetoxy group at C-10 using lead tetraacetate and BF_3_ etherate produceds 8-Acetoxy-4-((acetoxy) acetyl)-1 H-2-benzopyran-1-one. The protecting acetyl groups are then detached with *Pseudomonas fluorescens* lipase in a final hydrolysis step to afford oosponol (Kovács, and Sonnenbichler, 1997).

**Fig. 7:**
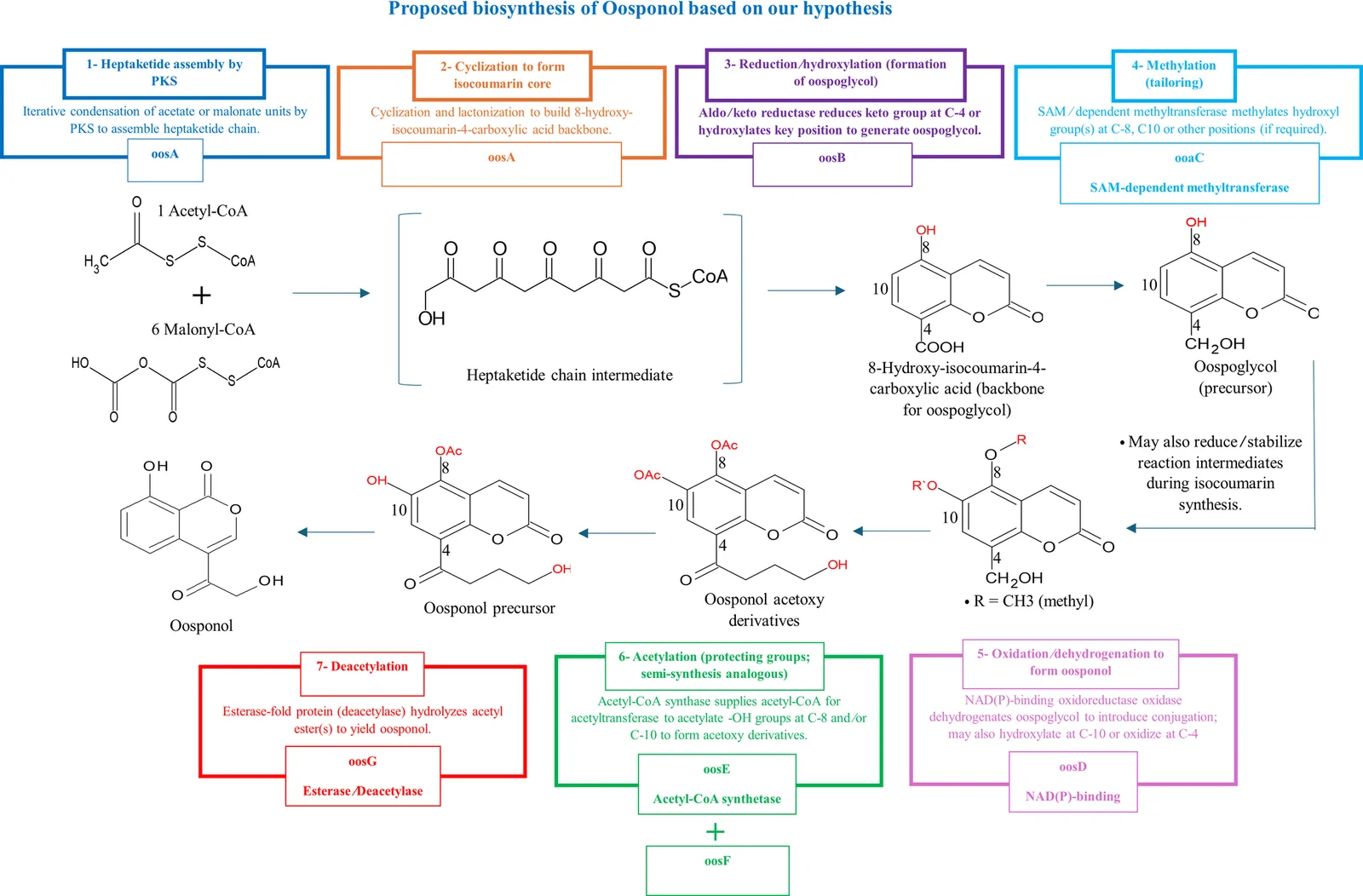
Demonstrates the proposed biosynthetic pathway of oosponol in *G. trabeum*. The pathway starts with the assembly of a heptaketide backbone by an iterative PKS (oosA), followed by cyclization, reduction, methylation, oxidation, acetylation, and final deacetylation mediated by the predicted enzymes oosB–oosG to yield oosponol. Gene assignment is basically inferred based on the high homologous protein sequences and domain structure or function of alternariol, citrinin and clavatol biosynthesis (Chooi et al. 2015; Hajjaj et al., 1999)

In agreement with this biosynthetic framework, our phylogenetic analysis revealed that one of the *G. trabeum* PKS enzymes namely PKS8-1, shared high sequence similarity with PKS enzymes associated with the biosynthesis of alternariol, clavatol and citrinin (Bailey and Elkan 1994). Nevertheless, the biosynthetic pathway of oosponol and its derivatives remains unidentified. Although several intermediates have been isolated from the fermentation culture of the related species (*Gloeophyllum abietinum*), none of the enzymes that directly involved in the biosynthesis of these intermediates have been identified. Therefore, our proposed assignment (Fig. 7) between the synthesis steps and the corresponding biosynthetic pathways is largely inferred based on the high homologous protein sequences and domain structure or function of alternariol, citrinin and clavatol biosynthesis genes. For example, an investigation by (Chooi et al. 2015) on the biosynthesis of alternariol, revealed that a single polyketide gene (SnPKS19) is sufficient to biosynthesize the heptaketide-based dibenzopyranone compound in *Parastagonospora nodorum*. Interestingly, although the SnPKS19 gene was surrounded with additional tailoring genes, including two o-methyltransferase, a *β*-lactamase, a short chain dehydrogenase, a regulatory protein and a transporter, transcription data showed that only SnPKS19 and one of the o-methyltransferase were expressed during microarray experiments (Ipcho et al. 2012). In light of these findings, it is plausible that few or one enzyme is sufficient for the biosynthesis of alternariol compound, providing a useful model for predicting the biosynthetic machinery involved in isocoumarin based compounds.

Similarly, (Hajjaj et al. 1999) found that, the biosynthesis of citrinin starts with the condensation of one acetyl-CoA and three malonyl-CoA to a tetraketide backbone, followed by a series of subsequent tailoring reactions (e.g. oxidation and reduction). In comparison, our predicted PKS8-1 biosynthetic gene cluster consists of the following genes: polyketide beta-ketoacyl-synthase, NAD P-binding protein, galactose mutarotase-like protein, NAD P-binding protein, acetyl-CoA synthetase-like protein, MFS general substrate transporter, MFS general substrate transporter, acid protease, Aldo/keto reductase, and SAM dependent methyltransferase.

However, the initial antiSMASH prediction indicated the absence of SAM dependent methyltransferase whereas subsequent manual genome annotation identified a putative SAM-dependent methyltransferase within scaffold 8, using Artemis and Blast search. This discrepancy is likely because the sequence similarity, domain architecture, or genomic context of this gene, did not satisfy the software’s criteria for inclusion within the cluster. Moreover, incomplete or fragmented genome assemblies may lead to missing or fragmented genes or BGC boundaries (Blin et al. 2021). Based on the organisation of the predicted BGC, and the similarities with previously characterised fungal polyketide pathways, we therefore, hypothesise that the biosynthesis of oosponol initiates with the iterative condensation of acetate or malonate units by the polyketide β-ketoacyl synthase (PKS) to assemble the heptaketide chain. This backbone then undergoes subsequent cyclization to build the 8-hydroxy-isocoumarin-4-carboxylic acid (the backbone for the oosponol precursor-oospoglycol) (Atanasoff-Kardjalieff et al. 2023). Following the formation of this intermediate, the aldo/keto reductase hypothetical role is either reduces keto group at C-4 or hydroxylates key position to generate oospoglycol. Moreover, it might participate in the reduction of stabilization reactive intermediates during isocoumarin synthesis (Hintzpeter et al. 2015). Thereafter, the SAM-dependent methyltransferase likely plays a tailoring role by methylating hydroxyl groups at C-8, C-10, or other positions on the precursor-oospoglycol (Ma et al. 2014). In the subsequent oxidation step, the NAD P-binding protein is supposed to mediate the oxidation or dehydrogenation of oospoglycol into oosponol, via the introduction of conjugation into rings or side chains to improve the bioactivity. Furthermore, it might catalyse the hydroxylation at positions like C-10 or oxidation at C-4 (Shimizu et al. 2005). As for the protein encoding acetyl-CoA synthetase, we hypothesized its role as a local supplier of acetyl CoA for the acetyltransferase activity, which potentially acetylate the hydroxyl groups at C-8 and C-10, to form acetoxy derivatives as seen in the semi-synthesis. Alternatively, it may co-catalyse with a trans-acting acetyltransferase (Campbell and Vederas 2010). Although, protease like enzymes are rarely involved in PKS like compound synthesis, some esterase-fold genes can facilitate the deacetylation ⁄ hydrolytic cleavage reactions, mimicking the *Pseudomonas fluorescens* lipase used in the semi-synthesis, to hydrolyse the acetyl ester in the final step of the biosynthesis (Barriuso et al. 2013). There is also the MFS gene, and its hypothetical function is the export of oosponol or its derivatives out of the cell, to avoid intracellular toxicity (Alouane et al. 2021). The final gene in the predicted BGC is galactose mutarotase-like protein, and its putative role is to regulate or redox-balancing function, for example it could act on NADPH, a non-canonical epimerase involved in ring rearrangements or chiral centre stabilizations (Thoden et al. 2003). Notable, some genes within the above predicted BGC may be transcriptionally silent or show no expression under standard laboratory cultivation conditions (Ipcho et al. 2012). Nevertheless, our rationale linkage between the predicted BGC and the characterised isocoumarin compounds (e.g. oospoglycol and oosponol) from *G. trabeum* is based mainly on the identification of fungal polyketide synthases (PKSs) from this species, phylogenetic similarity of these predicted PKS with known biologically active polyketides genes, the presence of the required tailoring enzymes for coumarin precursors modifications, the consistency of the predicted biosynthetic pathway with established polyketide derivatives correlated to non-reducing polyketide compounds, and finally, the chemical structure of the oospoglycol and oosponol which supports a polyketide origin.

## Conclusion

In this study, we provide a valuable perception into the metabolic potential of a wood-decay basidiomycetes *G. trabeum*, underscoring its ability to produce atypical, specialized metabolites (e.g. coumarins). Furthermore, genome analysis allowed the prediction of several BGCs, particularly those associated with isocoumarin derived compounds, and further highlighted the fungus’s potential for novel natural products biosynthesis. Complementing these genomic findings, the molecular docking analysis, demonstrated that our tested compounds particularly, oosponol and oospoglycol, have promising antibiotic potentials, through their strong interaction with multiple target proteins including FtsZ and UspA1. Although control ligands consistently exhibited stronger binding energies and better interactions, our test compounds offered favourable solubility and ADMET properties, especially in cases like FabI and GmhA target proteins. Moreover, both hydrogen bonding and hydrophobic affinity engaged in their interactions, with noteworthy flexibility in acclimating to different binding pockets. As well as their diverse interactions modes observed (particularly oospoglycol) across other microbes suggest its potential as a broad-spectrum antimicrobial agent, specifically if modified to enhance some of its current suboptimal binding affinity through structural modification (e.g. C-6 halogenation (Cl/F), 7-O-alkylation (methyl or prenyl)). Such pharmacokinetic properties may help in compensating the slightly lower binding affinities, mostly during the early stages of drug optimisation. Taken together, our docking analysis underscored the worthiness of harmonizing binding affinity of a potential drug with its pharmacokinetic properties and further agree with previous studies reported the importance of integrating drug-like properties with its structure based-design. Beyond the docking analysis, our phylogenetic tree analysis revealed the diversity of the PKS enzymes of this organism and emphasised their evolutionary adaptation to catalyse the biosynthesis of distinct types of polyketide-based molecules. Finally, giving that *G. trabeum* is the only species within the Gloeophyllales order with an available genomic data, our findings position this fungus as a talented species for future synthetic biology applications designed to develop novel and sustainable antibiotics.

### Future studies recommendation

Although our genome mining, phylogenetic analyses, and manual annotation provide strong evidence supporting the identification of the putative oosponol biosynthetic gene cluster, we acknowledge that functional validation remains necessary. Future studies employing transcriptomic approaches, such as RT-qPCR or RNA-seq under oosponol-producing conditions, together with targeted gene disruption or knockout experiments, will be essential to confirm the involvement of the proposed genes in oosponol biosynthesis and establish a direct causal relationship between gene expression and metabolite production. 

## Supplementary Information

Below is the link to the electronic supplementary material.

Supplementary Material 1 (DOCX 75.5 MB)

## Data Availability

All genomic datasets given or analysed in this study are included in the manuscript and its supplementary materials. The NMR shifts of compound 1 were deposited in BMRBig under the deposition ID = 53776. Further supporting background is available from the corresponding authors upon request.
