# Supplementary material for: Exploring rare coumarins biosynthesis by Gloeophyllum trabeum: an integrated computational genomics, molecular docking and spectroscopic approach
Source: World J Microbiol Biotechnol. 2026 Aug 14;42(9):465. doi: 10.1007/s11274-026-05181-9 (PMC13476290; doi:10.1007/s11274-026-05181-9)
Supplement: Supplementary file 1 [file 11274_2026_5181_MOESM1_ESM.docx]

**Exploring rare coumarins biosynthesis by *Gloeophyllum trabeum*: an integrated computational genomics, molecular docking and spectroscopic approach**

**Suhad A. A. Al-Salihi^1*^, Trong Tuan Dao^2^, Saipul Maulana^3^, Andy M. Bailey^4*^**

^1^ Department of Biotechnology, College of Applied Sciences, University of Technology, Baghdad, Iraq.

^2^ School of Pharmacy, University of Medicine and Pharmacy at Ho Chi Minh City, Vietnam.

^3^ Department of Pharmacy, Faculty of Mathematics and Natural Sciences, Universitas Tadulako, Kota Palu, Indonesia.

^4^ School of Biological Sciences, University of Bristol, Bristol, UK.

**^*^** Corresponding authors: [suhad.a.ahmed@uotechnology.edu.iq](mailto:suhad.a.ahmed@uotechnology.edu.iq), [Andy.Bailey@bristol.ac.uk](mailto:Andy.Bailey@bristol.ac.uk)

**Supplementary Data**

**Table of content:**

| Content | | Page |
| --- | --- | --- |
| 1. | 1. Materials & Methods | 2 |
| 2. | 1.1. Microbial suspension preparation | 3 |
| 3. | 1.2. Metabolites preparation for HPLC-MS analysis | 3 |
| 4. | 2. Results | 3 |
|  | Table S1: Docking-based evaluation of the test compound and benchmark ligands across microbial protein targets | 5 |
| 5. | 2.1. Chemical characterisation of isolated compounds | 7 |
| 6. | Figure S1: Key HMBC (H🡪C) correlations for compound 1 | 7 |
| 7. | Table S2: ^1^H (500 MHz) and ^13^C (125 MHz) NMR Data of 1*^a^* and 4 | 8 |
| 8. | Figure S2: UV/vis (DAD) spectrum of 1. | 8 |
| 9. | Figure S3: IR spectrum of 1 | 9 |
| 10. | Figure S4: (-) ESI-MS spectrum of 1. | 9 |
| 11. | Figure S5: ^1^H NMR spectrum (acetone-*d*_6_, 500 MHz) of 1. | 10 |
| 12. | Figure S6: ^13^C NMR spectrum (acetone-*d*_6_, 125 MHz) of 1. | 10 |
| 13. | Figure S7: COSY spectrum of 1. | 11 |
| 14. | Figure S8: HSQC spectrum of 1. | 11 |
| 15. | Figure S9: HMBC spectrum of 1. | 12 |
| 16. | Figure S10: (-)ESI-MS spectrum of 2. | 12 |
| 17. | Figure S11: ^1^H NMR spectrum (CD_3_OD, 500 MHz) of 2. | 13 |
| 18. | Figure S12: ^13^C NMR spectrum (CD_3_OD, 125 MHz) of 2 | 13 |
| 19. | Figure S13: (-)ESI-MS spectrum of 3 | 14 |
| 20. | Figure S14: ^1^H NMR spectrum (CD_3_OD, 500 MHz) of 3. | 14 |
| 21. | Figure S15: ^13^C NMR spectrum (CD_3_OD, 125 MHz) of 3. | 15 |
| 22. | Figure S16: UV/vis (DAD) spectrum of 4 | 15 |
| 23. | Figure S17: IR spectrum of 4 | 16 |
| 24. | Figure S18: (+)ESI-MS spectrum of 4. | 16 |
| 25. | Figure S19: ^1^H NMR spectrum (CD_3_OD, 500 MHz) of 4 | 17 |
| 26. | Figure S20: ^13^C NMR spectrum (CD_3_OD, 125 MHz) of 4 | 17 |
| 27. | Figure S21: HSQC spectrum of 4 | 18 |
| 28. | Figure S22: HMBC spectrum of 4 | 18 |
| 29. | S23: Illustration of 2D (panel A) and 3D (panel B) ligand–protein interactions of native ligand (C_19_H_2_6N_2_O_2_) and candidate compounds; compound 1, oospoglycol & oosponol with target protein (MurG) of *B. pseudomallei* respectively | 19 |
| 30. | Figure S24: Illustration of 2D (panel A) and 3D (panel B) ligand–protein interactions of native ligand (Mut148233) and candidate compounds; compound 1, oospoglycol & oosponol with target protein (GmhA) of *B. pseudomallei* respectively. | 20 |
| 31. | Figure S25: Illustration of 2D (panel A) and 3D (panel B) ligand–protein interactions of native ligand (1-hydroxy-2,3,1-benzodiazaborinine-2(1H)-carbothioamide) and candidate compounds; compound 1, oospoglycol & oosponol with target protein (FabI) of *E. coli* respectively. | 21 |
| 32. | Figure S26: Illustration of 2D (panel A) and 3D (panel B) ligand–protein interactions of native ligand (1-hydroxy-2,3,1-benzodiazaborinine-2(1H)-carbothioamide) and candidate compounds; compound 1, oospoglycol & oosponol with target protein (FtsZ) of *E. coli* respectively. | 22 |
| 33. | Figure S27: Illustration of 2D (panel A) and 3D (panel B) ligand–protein interactions of native ligand (Triclosan) and candidate compounds; compound 1, oospoglycol & oosponol with target protein (FabI) of *M. catarrhalis* respectively. | 23 |
|  | Figure S28: Illustration of 2D (panel A) and 3D (panel B) ligand–protein interactions of native ligand (Ceftobiprole) and candidate compounds; compound 1, oospoglycol & oosponol with target protein (*UspA1*) of *M. catarrhalis* respectively. | 24 |
|  | Figure S29: Illustration of 2D (panel A) and 3D (panel B) ligand–protein interactions of native ligand (Triclosan) and candidate compounds; compound 1, oospoglycol & oosponol with target protein (FabI) of *N. meningitidis* respectively*.* | 25 |
|  | Figure S30: Illustration of 2D (panel A) and 3D (panel B) ligand–protein interactions of native ligand (Triclosan) and candidate compounds; compound 1, oospoglycol & oosponol with target protein (FabI) of *S. aureus* respectively | 26 |
|  | Figure S31: Illustration of 2D (panel A) and 3D (panel B) ligand–protein interactions of native ligand (Lumacaftor) and candidate compounds; compound 1, oospoglycol & oosponol with target protein (FemX) of *S. aureus* respectively. | 27 |
|  | Figure S32: Illustration of 2D (panel A) and 3D (panel B) ligand–protein interactions of native ligand (((2~{R})-3-methyl-2-[(~{E})-[2-methyl-3-oxidanyl-5-(phosphonooxymethyl)pyridin-4-yl]methylideneamino]-3-sulfanyl-butanoic acid)) and candidate compounds; compound 1, oospoglycol & oosponol with target protein (SufS) of *Bacillus subtilis* respectively. | 28 |
|  | Figure S33: RMSD profiles of eight receptor–ligand complexes during a 100 ns molecular dynamics simulation. |  |
| 34. | Table S2: Predicted BGCs using FungiSmash platform. | 29 |
|  | Figure S29: Alignment of motif sequences and their conserved positions in alternariol, PKS8-1, clavatol and citrinin. | 30 |
| 35. | Figure S28: Gene function annotation of the predicted T1PKS-like BGCs, using AntiSMASH, Softberry, Artemis, and Local BLAST platform. A- scaffold-8, B- scaffold-11, C- scaffold-12, D- scaffold-13, E- scaffold-14, F- scaffold-16, G- scaffold-24. | 31 |
| 36. | References | 32 |

**1. Materials and Methods**

**1.1. Microbial suspension preparation**

A method described by Hartley et al. (2009) was used to prepare B. subtilis (strain ATCC6633) suspension. To activate the spores and kill the vegetative cells, the obtained spore suspension was immersed in a water bath at 70 °C for 30 min and subsequently stored at 4 °C until use.

While Escherichia coli overly plates were prepared by inoculating 5 ml of Luria-Bertani (LB: 10 g/L tryptone, 10 g/L NaCl, 10 g/L yeast extract and pH 7) with a fresh single colony of DH5α plate and shaken at 37°C overnight. The grown culture was then used to subculture 100 ml of sterile LB and further shaken at 37°C to reach optical density OD600 = 1.0. 2 ml of this culture plus 0.4% of 2,3,5-triphenyl-2H-tetrazolium chloride (TTC) were added to 1 L of molten LB agar (approximately 50°C) before pouring into plates.

Similarly to E. coli overly preparation, *Saccharomyces services* overly plates were prepared through the inoculation of a single colony of strain Y10000 and shaken at 28°C overnight. This culture was then added to 250 ml Erlenmeyer flask containing 100 ml of YPD broth (10 g/L of yeast extract, 20 g/L of peptone and 20 g/L of D-glucose) to be shaken at 28°C until reach optical density OD600 = 0.1. The obtained culture was then used to prepare the overly plates as described above.

**1.2. Metabolites preparation for HPLC-MS analysis**

Following the selection of the appropriate solvent system for compound separation, column chromatography was performed to isolate potential biologically active metabolites from the fermentation culture of G. trabeum. According to (Al-Salihi et al., 2019), a cylindrical glass tube with a stopcock at the end was used to prepare the column. Then a small amount of glass wool was placed at the bottom to prevent eluent leakage. Enough silica gel (Sephadex LH-20, MCI gel CHP 20P) was packed into the column, leaving a 5-10 cm space at the top for solvent addition. The column was held vertically using a ring stand with three clamps. To pre-elute the column, 100 ml of methanol was gently added on top of the adsorbent, ensuring the silica surface remained flat. The stopcock was then opened to drain the solvent, leaving 1 cm above the packed silica to prevent it from drying out. After setting up the column, 1 ml of 200-500 mg of crude extract was gently added to the sides of the column using a Pasteur pipette. The polarity of the eluent (methanol) was gradually increased by mixing it with different ratios of deionized water (30%, 20%, 10%, 5%, and 0%) before adding 100 ml each time. The stopcock was opened to allow the eluent to flow through the silica, facilitating compound separation. The fractions were collected in clean, dry test tubes, and their purity was further analyzed using LC-MS on a Waters 2767 HPLC system, equipped with a Waters 2545 pump system and a Phenomenex LUNA C18 column (2.6 μm, 100 Å, 4.6 x 100 mm) along with a Phenomenex Security Guard precolumn Luna C5 300 Å for reverse-phase chromatography analysis. A gradient solvent system was employed: solvent A (water with 0.05% formic acid) and solvent B (acetonitrile with 0.045% formic acid). The gradient program was as follows: 0 minutes, 10% B; 15 minutes, 90% B; 16 minutes, 95% B; 17 minutes, 95% B; 18 minutes, 10% B; 20 minutes, 10% B, with a flow rate of 1 ml/min. Compound UV absorbance was detected between 200 nm and 400 nm using a Waters 2998 diode array detector. When appropriate, a Waters 2424 Evaporative Light Scattering Detector (ELSD) measured the metabolite amounts. An HPLC preparative instrument was also used to collect eluents with specified separation retention times and UV/ELSD activity. The Waters mass-directed auto-purification system, equipped with a Waters 2767 auto-sampler and Waters 2545 pump system, was used for reverse-phase chromatography purification with a Phenomenex LUNA column (5 μm, C18, 100 Å, 10 x 250 mm) and a Phenomenex Security Guard precolumn Luna C5 300 Å, eluted at 16 ml/min (Al-Salihi et al., 2019).

**2. Results**

**Table S1: Docking-based evaluation of the test compound and benchmark ligands across microbial protein targets**

| **Microorganism** | **Compound** | **Targeted Protein** | **Binding Energy (kcal/mol)** |
| --- | --- | --- | --- |
| 1. *Burkholderia pseudomallei* | 5-(4-amino-2-methylphenoxy)-2-hexyl-4-hydroxy-1-methylpyridinium (C19H26N2O2, ID = 73437603) | MurG  (PDB ID = 4BKU) | -7.0915 |
|  | Compound 1 |  | -5.4622 |
|  | Oospoglycol |  | -5.5930 |
|  | Oosponol |  | -5.3418 |
|  | Mut148233 | GmhA  (PDB ID = 8V4J) | -6.3196 |
|  | Compound 1 |  | -4.4843 |
|  | Oospoglycol |  | -4.6447 |
|  | Oosponol |  | -4.7535 |
| 2. *Escherichia coli* | 1-hydroxy-2,3,1-benzodiazaborinine-2(1H)-carbothioamide | FabI  (ID = 5CG2) | -4.7897 |
|  | Compound 1 |  | -5.2007 |
|  | Oospoglycol |  | -4.8190 |
|  | Oosponol |  | -5.0337 |
|  | Berberine | FtsZ  (ID = 1FSZ) | -5.6764 |
|  | Compound 1 |  | -5.1360 |
|  | Oospoglycol |  | -5.7261 |
|  | Oosponol |  | -5.5290 |
| 3. *Moraxella catarrhalis* | Triclosan (ID = 5564) | FabI  (ID = 7FCM) | -5.5159 |
|  | Compound 1 |  | -5.6071 |
|  | Oospoglycol |  | -5.6301 |
|  | Oosponol |  | -5.4138 |
|  | Ceftobiprole | UspA1  (PDB ID = 3ntn) | -5.8829 |
|  | Compound 1 |  | -5.0197 |
|  | Oospoglycol |  | -11.713 |
|  | Oosponol |  | -13.552 |
| 4. *Neisseria meningitidis* | Triclosan | FabI  (ID = 4M89) | -5.9342 |
|  | Compound 1 |  | -5.5706 |
|  | Oospoglycol |  | -5.3712 |
|  | Oosponol |  | -5.3181 |
| 5. *Staphylococcus aureus* | Triclosan | FabI  (ID = 3GR6) | -5.8284 |
|  | Compound 1 |  | -5.6214 |
|  | Oospoglycol |  | -5.4800 |
|  | Oosponol |  | -5.3962 |
|  | Lumacaftor | FemX  (ID = 6SNR) | -7.0188 |
|  | Compound 1 |  | -5.6206 |
|  | Oospoglycol |  | -5.7059 |
|  | Oosponol |  | -5.7775 |
| 6- *Bacillus subtilis* | ((2~{R})-3-methyl-2-[(~{E})-[2-methyl-3-oxidanyl-5-(phosphonooxymethyl)pyridin-4-yl]methylideneamino]-3-sulfanyl-butanoic acid)) | SufS with L-penicillamine  (ID = 7XEN) | -8.5319 |
|  | Test compound |  | -5.4501 |
|  | Oospoglycol |  | -5.6409 |
|  | Oosponol |  | -5.5165 |

**2.1. Chemical characterisation of isolated compounds**

The crude extract was subjected to column chromatography over silica gel RP-C18 (MeOH/H_2_O) and further purified by preparative LCMS (Luna 5μ C18 100 Å, 250 x 10 mm, 8 ml/min) using a 20 min 10-95% acetonitrile in water (+ 0.1 % formic acid) programme to afford **oospoglycol (2)** (Sonnenbichler et al., 1997) **, oosponol (3)** (Kovacs et al., 1997) **and FD48a (4)** (Sonnenbichler et al., 1989) together with one unknown iso-coumarin derivative **1**.

**Compound 1:** pale yellow solid; UV/vis (DAD) λmax nm: 358, 263, 228; IR *ν*max 3488, 2950, 1658, 1452, 1232 cm–1; δH (500 MHz, acetone-*d*6) 7.37 (1H, s, H-3), 7.35 (1H, d, *J* = 8.0, H-6), 7.24 (1H, d, *J* = 8.0 Hz, H-5), 4.92 (1H, m, H-9), 3.83 (1H, m, H-10a), 3.67 (1H, m, H-10b); δC (125 MHz, acetone-*d*6) 167.7 (C-1), 149.9 (C-8), 145.5 (C-7), 141.1 (C-3), 128.3 (C-

4a), 124.5 (C-6), 119.9 (C-4), 114.9 (C-5), 107.9 (C-8a), 70.4 (C-9), 66.8 (C-10).

**Compound 2:** yellow solid; δH (500 MHz, CD3OD) 7.67 (1H, t, *J* = 8.0, H-6), 7.46 (1H,s, H-3), 7.18 (1H, d, *J* = 8.0 Hz, H-5), 6.95 (1H, d, *J* = 8.0 Hz, H-7), 4.90 (1H, m, H-9), 3.86 (1H, m, H-10a), 3.70 (1H, m, H-10b); δC (125 MHz, CD3OD) 167.6 (C-1), 163.0 (C-8), 143.8 (C-3), 138.4 (C-6), 137.2 (C-4a), 119.7 (C-4), 116.3 (C-5), 114.3 (C-7), 107.3 (C-8a), 70.2 (C-9), 66.4 (C-10). The data are consistent with those reported for oospoglycol1

.

4**Compound 3:** pale yellow solid; δH (500 MHz, CD3OD) 8.31 (1H, s, H-3), 8.03 (1H, d,*J* = 8.0, H-5), 7.76 (1H, t, *J* = 8.0 Hz, H-6), 7.08 (1H, d, *J* = 8.0 Hz, H-7), 4.68 (2H, s, H-10); δC (125 MHz, CD3OD) 197.3 (C-9), 164.6 (C-1), 161.6 (C-8), 151.7 (C-3), 137.7 (C-6), 133.4 (C-4a), 116.1 (C-5), 115.7 (C-7), 106.1 (C-8a), 65.7 (C-10). The data are consistent with those reported for oosponol2

.

**Compound 4:** pale yellow solid; UV/vis (DAD) λmax nm: 339, 257, 228; IR *ν*max 3445, 2945, 1691, 1443, 1273 cm–1; δH (500 MHz, CD3OD) 7.70 (1H, t, *J* = 8.0, H-6), 7.26 (1H, d, *J* = 8.0 Hz, H-5), 6.95 (1H, d, *J* = 8.0 Hz, H-7), 4.67 (2H, s, H-9), 2.40 (3H, s, 3-CH3); δC (125 MHz, CD3OD) 167.5 (C-1), 162.9 (C-7), 154.4 (C-3), 139.4 (C-6), 138.6 (C-4a), 115.4 (C-7), 114.9 (C-4), 114.5 (C-5), 107.1 (C-8a), 57.5 (C-9), 16.8 (3-CH3). The 1H NMR data are consistent with those reported for FD48a3, here we first show 13C and 2D NMR data for this compound.

**Figure S1:** Key HMBC (H🡪C) correlations for compound **1**.

**Table S2:** ^1^H (500 MHz) and ^13^C (125 MHz) NMR Data of **1***^a^* and **4***^b^*

| Position | **1** | |  | **4** | | |
| --- | --- | --- | --- | --- | --- | --- |
|  | δ_H_ mult. (*J* in Hz) | δ_C_ |  | δ_H_ mult. (*J* in Hz) | δ_C_ |  |
|  |  |  |  |  |  |  |
| 1 |  | 167.7 |  |  | 167.5 |  |
| 3 | 7.37 s | 141.1 |  |  | 154.4 |  |
| 4 |  | 119.9 |  |  | 114.9 |  |
| 4a |  | 128.3 |  |  | 138.6 |  |
| 5 | 7.24 d (8.0) | 114.9 |  | 7.26 d (8.0) | 114.5 |  |
| 6 | 7.35 d (8.0) | 124.5 |  | 7.70 t (8.0) | 139.4 |  |
| 7 |  | 145.5 |  | 6.95 d (8.0) | 115.4 |  |
| 8 |  | 149.9 |  |  | 162.9 |  |
| 8a |  | 107.9 |  |  | 107.1 |  |
| 9 | 4.92 m | 70.4 |  | 4.67 s | 57.5 |  |
| 10 | 3.83 m | 66.8 |  |  |  |  |
|  | 3.67 m |  |  |  |  |  |
| 3-CH_3_ |  |  |  | 2.40 s | 16.8 |  |
|  |  |  |  |  |  |  |

*^a^* Recorded in acetone-*d*_6_. *^b^* Recorded in CD_3_OD.

**Figure S2:** UV/vis (DAD) spectrum of **1**.

**Figure S3.** IR spectrum of **1**.

**Figure S4.** (-)ESI-MS spectrum of **1**.

**Figure S5:** ^1^H NMR spectrum (acetone-*d*_6_, 500 MHz) of **1**.

**Figure S6:** ^13^C NMR spectrum (acetone-*d*_6_, 125 MHz) of **1**.

**Figure S7:** COSY spectrum of **1**.

**Figure S8:** HSQC spectrum of **1**.

**Figure S9:** HMBC spectrum of **1**.

**Figure S10.** (-)ESI-MS spectrum of **2**.

**Figure S11:** ^1^H NMR spectrum (CD_3_OD, 500 MHz) of **2**.

**Figure S12:** ^13^C NMR spectrum (CD_3_OD, 125 MHz) of **2**.

**Figure S13.** (-)ESI-MS spectrum of **3**.

**Figure S14:** ^1^H NMR spectrum (CD_3_OD, 500 MHz) of **3**.

**Figure S15:** ^13^C NMR spectrum (CD_3_OD, 125 MHz) of **3**.

**Figure S16:** UV/vis (DAD) spectrum of **4**.

**Figure S17.** IR spectrum of **4**.

**Figure S18.** (+)ESI-MS spectrum of **4**.

**Figure S19:** ^1^H NMR spectrum (CD_3_OD, 500 MHz) of **4**.

**Figure S20:** ^13^C NMR spectrum (CD_3_OD, 125 MHz) of **4**.

**Figure S21:** HSQC spectrum of **4**.

**Figure S22:** HMBC spectrum of **4**.

**A1**

**B1**

**A2**

**A3**

**A4**

**B3**

**B2**

**B4**

S23: Illustration of 2D (panel A) and 3D (panel B) ligand–protein interactions of native ligand (C_19_H_2_6N_2_O_2_) and candidate compounds; compound 1, oospoglycol & oosponol with target protein (MurG) of *B. pseudomallei* respectively.

**A1**

**B1**

**A2**

**B2**

**A3**

**B3**

**A4**

**B4**

Figure S24: Illustration of 2D (panel A) and 3D (panel B) ligand–protein interactions of native ligand (Mut148233) and candidate compounds; compound 1, oospoglycol & oosponol with target protein (GmhA) of *B. pseudomallei* respectively.

**A1**

**B1**

**A2**

**B2**

**A3**

**B3**

**A4**

**B4**

Figure S25: Illustration of 2D (panel A) and 3D (panel B) ligand–protein interactions of native ligand (1-hydroxy-2,3,1-benzodiazaborinine-2(1H)-carbothioamide) and candidate compounds; compound 1, oospoglycol & oosponol with target protein (FabI) of *E. coli* respectively.

**A1**

**B1**

**A2**

**B2**

**A3**

**B3**

**A4**

**B4**

Figure S26: Illustration of 2D (panel A) and 3D (panel B) ligand–protein interactions of native ligand (1-hydroxy-2,3,1-benzodiazaborinine-2(1H)-carbothioamide) and candidate compounds; compound 1, oospoglycol & oosponol with target protein (FtsZ) of *E. coli* respectively.

**A2**

**B2**

**A1**

**B1**

**A3**

**B3**

**A4**

**B4**

Figure S27: Illustration of 2D (panel A) and 3D (panel B) ligand–protein interactions of native ligand (Triclosan) and candidate compounds; compound 1, oospoglycol & oosponol with target protein (FabI) of *M. catarrhalis* respectively.

**A2**

**B2**

**A1**

**B1**

**A3**

**B3**

**A4**

**B4**

Figure S28: Illustration of 2D (panel A) and 3D (panel B) ligand–protein interactions of native ligand (Ceftobiprole) and candidate compounds; compound 1, oospoglycol & oosponol with target protein (*UspA1*) of *M. catarrhalis* respectively.

**A2**

**B2**

**A1**

**B1**

**A3**

**B3**

**A4**

**B4**

Figure S29: Illustration of 2D (panel A) and 3D (panel B) ligand–protein interactions of native ligand (Triclosan) and candidate compounds; compound 1, oospoglycol & oosponol with target protein (FabI) of *N. meningitidis* respectively*.* .

**A2**

**B2**

**A1**

**B1**

**A3**

**B3**

**A4**

**B4**

Figure S30: Illustration of 2D (panel A) and 3D (panel B) ligand–protein interactions of native ligand (Triclosan) and candidate compounds; compound 1, oospoglycol & oosponol with target protein (FabI) of *S. aureus* respectively

**A2**

**B2**

**A1**

**B1**

**A3**

**B3**

**A4**

**B4**

Figure S31: Illustration of 2D (panel A) and 3D (panel B) ligand–protein interactions of native ligand (Lumacaftor) and candidate compounds; compound 1, oospoglycol & oosponol with target protein (FemX) of *S. aureus* respectively

**A2**

**B2**

**A1**

**B1**

**A3**

**B3**

**A4**

**B4**

Figure S32: Illustration of 2D (panel A) and 3D (panel B) ligand–protein interactions of native ligand (((2~{R})-3-methyl-2-[(~{E})-[2-methyl-3-oxidanyl-5-(phosphonooxymethyl)pyridin-4-yl]methylideneamino]-3-sulfanyl-butanoic acid)) and candidate compounds; compound 1, oospoglycol & oosponol with target protein (SufS) of *Bacillus subtilis* respectively.

Figure S33: RMSD profiles of eight receptor–ligand complexes during a 100 ns molecular dynamics simulation, demonstrating the structural stability and the conformational fluctuations of each complex over time. The RMSD values (Å) were calculated relative to the initial structure. The colored traces represent the following complexes: **orange**, *P. pseudomallei* GmhA–ligand (PDB ID: 8V4J); **light orange**, *P. aeruginosa* GmhA–ligand (PDB ID: 4KBQ); **olive**, *E. coli* FabI–ligand (PDB ID: 5CG2); **light blue**, *E. coli* FtsZ–ligand (PDB ID: 1FSZ); **cyan**, *M. catarrhalis* FabI–ligand (PDB ID: 7CMI); **blue**, *N. meningitidis* FabI–ligand (PDB ID: 4B89); **purple**, *S. aureus* FabI–ligand (PDB ID: 3GR6); and **magenta**, *S. aureus* FemX–ligand (PDB ID: 6SNR).

Table S3: Predicted BGCs using FungiSmash platform

| **Cluster Type** | | **Position** | **Most similar homologous known cluster** |
| --- | --- | --- | --- |
| 1. | Terpene | Scaffold 1: 187,621 to 236,521 |  |
| 2. | NRPS-like | Scaffold 2: 2,919,311 to 2,983,693 |  |
| 3. | Terpene | Scaffold 2: 2,235, 163 to 3,266,503 |  |
| 4. | NRPS-like | Scaffold 3: 322,953 to 474,857 |  |
| 5. | Terpene | Scaffold 3: 680,144 to 711,792 | +-O-cadinol |
| 6. | T1PKS, NRPS-like | Scaffold 3: 2,749,714 to 2, 825,964 |  |
| 7. | Terpene | Scaffold 4: 1,333,303 to 1,365,071 |  |
| 8. | Terpene | Scaffold 5: 1 to 26,469 |  |
| 9. | NRPS-like | Scaffold 5: 1,986, 818 to 2,098,581 |  |
| 10. | Terpene | Scaffold 7: 1,186,006 to 1,221,489 |  |
| 11. | Terpene | Scaffold 7: 1,402,194 to 1,433,651 |  |
| 12. | T1PKS, NRPS-like | Scaffold 8: 1 to 69,237 | Orsellinic acid |
| 13. | Terpene | Scaffold 8: 144,170 to 175,375 |  |
| 14. | NRPS-like | Scaffold 8: 1,345,805 to 1,409,596 |  |
| 15. | Terpene | Scaffold 10: 208,542 to 241,795 |  |
| 16. | Terpene | Scaffold 10: 1,154,287 to 1,185,203 |  |
| 17. | Terpene | Scaffold 11: 33,713 to 64,740 |  |
| 18. | NRPS | Scaffold 11: 182,891 to 248,812 |  |
| 19. | Terpene | Scaffold 11: 630,502 to 661,536 |  |
| 20. | NRPS-like | Scaffold 11: 1,284,762 to 1,348,900 |  |
| 21. | Terpene | Scaffold 11: 1,590,018 to 1,608,216 |  |
| 22. | Terpene | Scaffold 12: 54,036 to 85,243 |  |
| 23. | Terpene | Scaffold 12: 312, 431 to 343,676 |  |
| 24. | Terpene | Scaffold 12: 1,150,045 to 1,190,197 |  |
| 25. | Terpene | Scaffold 12: 1,223,638 to 1,255,080 |  |
| 26. | NRPS-like | Scaffold 13: 14,707 to 78,762 |  |
| 27. | Terpene | Scaffold 13: 593,580 to 627,152 |  |
| 28. | NRPS-like | Scaffold 13: 731,701 to 795,595 |  |
| 29. | T1PKS | Scaffold 13: 873, 122 to 943,935 |  |
| 30. | T1PKS | Scaffold 13: 944,312 to 1,012,761 |  |
| 31. | Terpene | Scaffold 14: 417,626 to 448,761 |  |
| 32. | T1PKS | Scaffold 14: 779,043 to 848,920 |  |
| 33. | Terpene | Scaffold 15: 830,964 to 875,448 |  |
| 34. | NRPS-like | Scaffold 16: 165,754 to 230,069 |  |
| 35. | T1PKS | Scaffold 16: 295,457 to 360,659 |  |
| 36. | T1PKS | Scaffold 16: 386,683 to 451,833 |  |
| 37. | NRPS-like | Scaffold 17: 1 to 41,805 |  |
| 38. | Terpene | Scaffold 19: 194,427 to 232,370 | Clavaric acid |
| 39. | T1PKS | Scaffold 24: 4,443 to 73,992 |  |
| 40. | Terpene | Scaffold 60: 1 to 7,939 |  |

Figure S34: Demonstrates alignment of motif sequences and their conserved positions in alternariol, PKS8-1, clavatol and citrinin (identified using the MEME Suite) suggesting shared enzymatic and structural roles in the biosynthesis of isocoumarin-like compounds.

**A**

**B**

**C**

**D**

**E**

**F**

**G**

**H**

Figure S35: Gene function annotation of the predicted T1PKS-like BGCs, using antiSMASH, Softberry, Artemis, and Local BLAST platform. A = scaffold 3.1, B = scaffold 3.3, C = scaffold 13.4, D = scaffold 13.5, E = scaffold 14.2, F = scaffold 16.2, G = scaffold 16.3, H = scaffold 24

**References**

1. Al-Salihi, S.A., Dao, T.T., Williams, K., Bailey, A.M. and Foster, G.D., 2019. The biogenetic origin of the biologically active naematolin of *Hypholoma* species involves an unusual sesquiterpene synthase. *Molecular Biotechnology*, *61*, pp.754-762.

2. Hartley, A.J., de Mattos-Shipley, K., Collins, C.M., Kilaru, S., Foster, G.D. and Bailey, A.M., 2009. Investigating pleuromutilin-producing *Clitopilus* species and related basidiomycetes. *FEMS Microbiology Letters*, *297*(1), pp.24-30.

3. Sonnenbichler, J; Bliestle, IM; Peipp, H; Holdenrieder, O. Secondary fungal metabolites and their biological activities, I. Isolation of antibiotic compounds from cultures of Heterobasidion annosum synthesized in the presence of antagonistic fungi or host plant cells. *Bio Chem Hoppe-Seyler*, 1989, Vol. 370, pp. 1295–1303.
